# Structural determinants of *Vibrio cholerae* FeoB nucleotide promiscuity

**DOI:** 10.1016/j.jbc.2024.107663

**Published:** 2024-08-14

**Authors:** Mark Lee, Kate Magante, Camilo Gómez-Garzón, Shelley M. Payne, Aaron T. Smith

**Affiliations:** 1 Department of Chemistry and Biochemistry, University of Maryland, Baltimore County, Baltimore, Maryland, USA; 2 Department of Molecular Biosciences, University of Texas at Austin, Austin, Texas, USA; 3 John Ring LaMontagne Center for Infectious Disease, University of Texas at Austin, Austin, Texas, USA

**Keywords:** Feo, iron, NTPase, X-ray crystallography, isothermal titration calorimetry

## Abstract

Ferrous iron (Fe^2+^) is required for the growth and virulence of many pathogenic bacteria, including *Vibrio cholerae* (*Vc*), the causative agent of the disease cholera. For this bacterium, Feo is the primary system that transports Fe^2+^ into the cytosol. FeoB, the main component of this system, is regulated by a soluble cytosolic domain termed NFeoB. Recent reanalysis has shown that NFeoBs can be classified as either GTP-specific or NTP-promiscuous, but the structural and mechanistic bases for these differences were not known. To explore this intriguing property of FeoB, we solved the X-ray crystal structures of *Vc*NFeoB in both the apo and the GDP-bound forms. Surprisingly, this promiscuous NTPase displayed a canonical NFeoB G-protein fold like GTP-specific NFeoBs. Using structural bioinformatics, we hypothesized that residues surrounding the nucleobase could be important for both nucleotide affinity and specificity. We then solved the X-ray crystal structures of N150T *Vc*NFeoB in the apo and GDP-bound forms to reveal H-bonding differences surrounding the guanine nucleobase. Interestingly, isothermal titration calorimetry revealed similar binding thermodynamics of the WT and N150T proteins to guanine nucleotides, while the behavior in the presence of adenine nucleotides was dramatically different. AlphaFold models of *Vc*NFeoB in the presence of ADP and ATP showed important conformational changes that contribute to nucleotide specificity among FeoBs. Combined, these results provide a structural framework for understanding FeoB nucleotide promiscuity, which could be an adaptive measure utilized by pathogens to ensure adequate levels of intracellular iron across multiple metabolic landscapes.

Iron (Fe) is an essential nutrient for nearly all lifeforms due to its use as a cofactor in numerous biochemical processes, including oxidative phosphorylation, *de novo* DNA synthesis, and nitrogen fixation, among others (1, 2, 3, 4, 5). To harness the power of this element, iron must first be acquired from the environment before it can be biologically incorporated into proteins and enzymes. For many organisms, including most bacteria, the prevalent environmental oxidation state of iron dictates its mode of acquisition. For example, the highly insoluble ferric iron (Fe^3+^) is prevalent in oxic environments, and bacteria will commonly deploy siderophores to solubilize and capture this form of iron. Membrane receptors then translocate the siderophore-chelated iron into the cytosol, where the iron is either removed by reductive dissociation or by cleavage of the siderophore (6, 7). Additionally, some pathogenic bacteria can sequester either free heme (iron protoporphyrin IX) or utilize hemophores to remove heme from host hemoproteins (*e.g.*, hemoglobin and myoglobin). Like siderophore-mediated uptake, membrane receptors then translocate the heme into the cytosol where the heme is either recycled or destroyed to remove the iron contained within (8, 9, 10, 11). In contrast, when living within anoxic or acidic environments, bacteria commonly encounter the more labile, but also more reactive, ferrous iron (Fe^2+^) (12, 13, 14, 15). Unfortunately, despite its strong contribution to bacterial metal homeostasis and pathogenesis, the mechanisms of bacterial Fe^2+^ acquisition are poorly understood compared to the mechanisms of Fe^3+^ uptake and heme acquisition.

While some auxiliary Fe^2+^ transport systems have been identified, the ferrous iron transport (Feo) system is the most widely distributed and conserved Fe^2+^ acquisition system across the prokaryotic domain (12, 13, 14, 15), although Feo’s precise mechanism of function remains unclear. Canonically, the *feo* operon encodes for three proteins, FeoA, FeoB, and FeoC (16), although FeoC is the least conserved of these proteins (13, 14, 15, 17) (Fig. 1). FeoA and FeoC are known to be small (*ca.* 7–10 kDa) cytosolic proteins, while FeoB is a large (*ca.* 80–100 kDa) polytopic transmembrane protein that contains an N-terminal soluble G-protein-like domain termed NFeoB (12, 14, 18). The status of GTP and GDP bound to the NFeoB region is hypothesized to control the opening and closing of the FeoB pore and therefore the translocation of Fe^2+^ into the bacterial cytosol (14, 15). The roles of FeoA and FeoC remain somewhat enigmatic; however, these proteins have been shown to interact with NFeoB *in vitro* (19), FeoA appears to regulate GTP hydrolysis *in vitro* (17), and some FeoCs bind oxygen-sensitive [Fe-S] clusters, presumably for regulatory purposes (20). *In vivo*, several observations indicate that both proteins interact with FeoB and are required for Feo-dependent iron uptake in *Vibrio cholerae* (*Vc*) (21, 22), the pathogenic bacterium responsible for the diarrheal disease cholera. Interestingly, bacterial two hybrid (BACTH) systems demonstrated that both *Vc*FeoA and *Vc*FeoC were found to interact with intact *Vc*FeoB in the cell (23), although the precise nature of this complex and its mechanism are still unclear.

**Figure 1:**
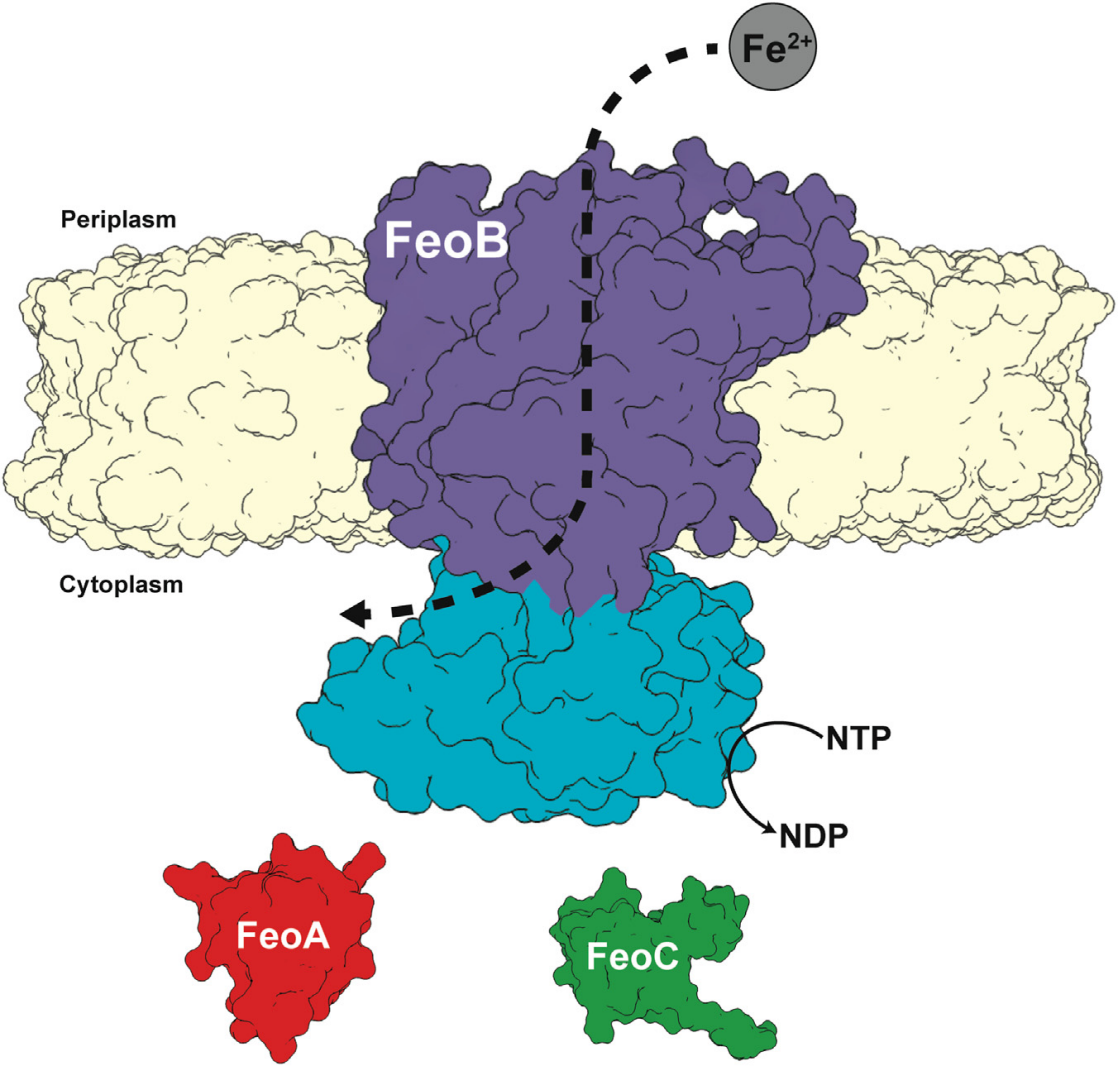
**Cartoon depiction of the tripartite *Vibrio cholerae* ferrous iron transport (Feo) system.** In *V. cholerae* the Feo system consists of three proteins: FeoA (colored in *red*) and FeoC (colored in *green*), both of which are cytosolic, and a polytopic transmembrane protein, FeoB (colored in *purple*), with a soluble N-terminal domain termed NFeoB (colored in *teal*). The NFeoB domain of *V. cholerae* FeoB was recently discovered to be nucleotide promiscuous and is best classified as an NTPase rather than a strict GTPase.

FeoB from *V. cholerae* was recently shown to hydrolyze ATP, GTP, and other NTPs *in vitro*, and this function can serve to supply *V. cholerae* with Fe^2+^ *in vivo*, indicating that this FeoB may be better classified as an NTPase rather than a strict GTPase (24, 25). This promiscuity for NTP consumption was then shown to occur *in vitro* for other FeoBs from a handful of infectious bacterial species such as *Helicobacter pylori, Streptococcus mutans, Staphylococcus aureus,* and *Bacillus cereus*, which could suggest that NTP promiscuity may be a common theme used by some pathogenic bacteria to acquire iron and to establish infection. NFeoB proteins contain generally conserved G-motifs that are common amongst G-proteins and are responsible for binding to different segments of the guanine nucleotide, with G1, G2, and G3 binding to the α-, β-, and γ-phosphates, while the G4 and G5 motifs interact with the nucleobase (26). Sequence analyses suggested that differentially conserved residues within the G4 and G5 motifs might be important for both guanine recognition and NTPase activity in several (N)FeoBs (27, 28). Specific to *V. cholerae* FeoB, variants analyzed in which the G5 motif residues Ser148 and Asn150 were altered (*i.e.*, S148V and N150T *Vc*NFeoB) were significantly attenuated in ATPase activity while minimal effects on GTPase activity were observed, indicating that these G5 residues may play a critical role in NTPase function *versus* GTPase function (24). However, the structural basis of nucleotide promiscuity in FeoB remained unknown, precluding a more comprehensive understanding of this unique aspect of the Feo system.

In this work, we have structurally and biophysically characterized the *Vc*NFeoB domain in order to understand its nucleotide promiscuity. Using X-ray crystallography, we determined the structures of apo and GDP-bound *Vc*NFeoB, which reveals a conserved NFeoB fold composed of a G-protein-like domain tethered to a GDI domain, despite the nucleotide promiscuous nature of *Vc*FeoB. Additionally, we determined the X-ray crystal structures of apo and GDP-bound N150T *Vc*NFeoB, and we show how differences in residues at the G5 motif alter the hydrogen-bonding interactions surrounding the guanine nucleobase. Isothermal titration calorimetry (ITC) was used to determine substrate affinities and stoichiometries of different nucleotides to both the wild-type and variant *Vc*NFeoBs. Intriguingly, we demonstrate dramatic differences in the behavior of the *Vc*NFeoB towards GDP/GMP-PNP binding compared to ADP/AMP-PNP binding, which could be rationalized using AlphaFold modeling. Taken together, these findings provide a structural framework for understanding the nucleotide promiscuity of *Vc*FeoB, which could be leveraged for future developments of targeted therapeutics to tackle issues of *V. cholerae* pathogenesis, as recently demonstrated (29).

## Results

### The VcNFeoB NTPase domain purifies as a nucleotide-free monomer that displays Broad NTPase activity

To prepare the *Vc*NFeoB NTPase domain for crystallization trials, we overproduced in *Escherichia coli* a previously designed construct that encodes for a non-cleavable (His)_6_-tagged version of the protein (*i.e.*, *Vc*NFeoB(His)_6_) (24). As this protein was not initially suitable for crystallization from immobilized metal-affinity chromatography (IMAC), we added two additional purification steps: anion exchange chromatography (AEX) and size-exclusion chromatography (SEC), both of which revealed interesting biophysical properties of *Vc*NFeoB(His)_6_. First, regarding AEX, we monitored the 260 nm/280 nm ratio through the entire chromatography process, and noted a normal value of ≈0.6, indicating that *Vc*NFeoB(His)_6_ does not co-purify with nucleotide, unlike previous reports of *E. coli* NFeoB overproduced in *E. coli* (30). Second, SEC of either crudely purified *Vc*NFeoB(His)_6_ (IMAC only) or polished *Vc*NFeoB(His)_6_ (after SEC) showed only the presence of a dominant monomeric species in solution (Fig. S1), consistent with our previous *in vitro* studies on Feo proteins (from *V. cholerae* and others) recombinantly produced in *E. coli*. It is possible that FeoA and/or FeoC may be necessary to induce FeoB oligomerization, and *in vivo* studies have suggested this to be the case for *V. cholerae* (23). However, other Feo systems appear to be functional monomers *in vitro*, and this highly pure, monomeric *Vc*NFeoB(His)_6_ domain was active against multiple nucleotide triphosphates, most notably ATP and GTP, as previously described (24); thus, the precise oligomeric state of FeoB remains controversial and requires further exploration. For all subsequent constructs (*vide infra*), a similar overproduction and purification process was employed, producing highly pure, homogeneous, and monomeric protein (Fig. S1).

### The structure of the apo VcNFeoB NTPase domain reveals a typical NFeoB fold

In order to characterize the three-dimensional structure of the *Vc*NFeoB NTPase domain, we sought to crystallize the *Vc*NFeoB(His)_6_ protein. Crystals of the apo domain were generated and ultimately diffracted to a modest 3.7 Å resolution in the *P*_1_ space group consistent with 8 molecules (dimer of tetramers) in the asymmetric unit (ASU) (Table S1). This oligomerization is likely crystallization-induced based on our in-solution studies (Fig. S1). We solved this structure by using molecular replacement coupled with model building and restrained refinement approaches (*R*_w_/*R*_f_ = 0.210/0.268) (Table S1), and an analysis of the crystal contacts suggested that the (His)_6_ tag may have impacted the crystal quality. To overcome this issue, *Vc*NFeoB was recloned into a vector encoding for N-terminal (His)_6_ tag fused to a cleavable SUMO moiety. Expression and purification followed the same procedures as previously described (*vide supra*), and the SUMO tag was cleaved prior to crystallization. Crystals of the SUMO-cleaved apo domain were generated and ultimately diffracted to 2.3 Å resolution in the *P*_121_ space group consistent with 2 molecules in the asymmetric unit (ASU) (Table S1). This oligomerization is likely crystallization-induced based on our in-solution studies (Fig. S1). Using the 3.7 Å resolution model, we were able to solve the 2.3 Å resolution structure of the tagless protein (*R*_w_/*R*_f_ = 0.203/0.266) (Table S1).

**Figure 2:**
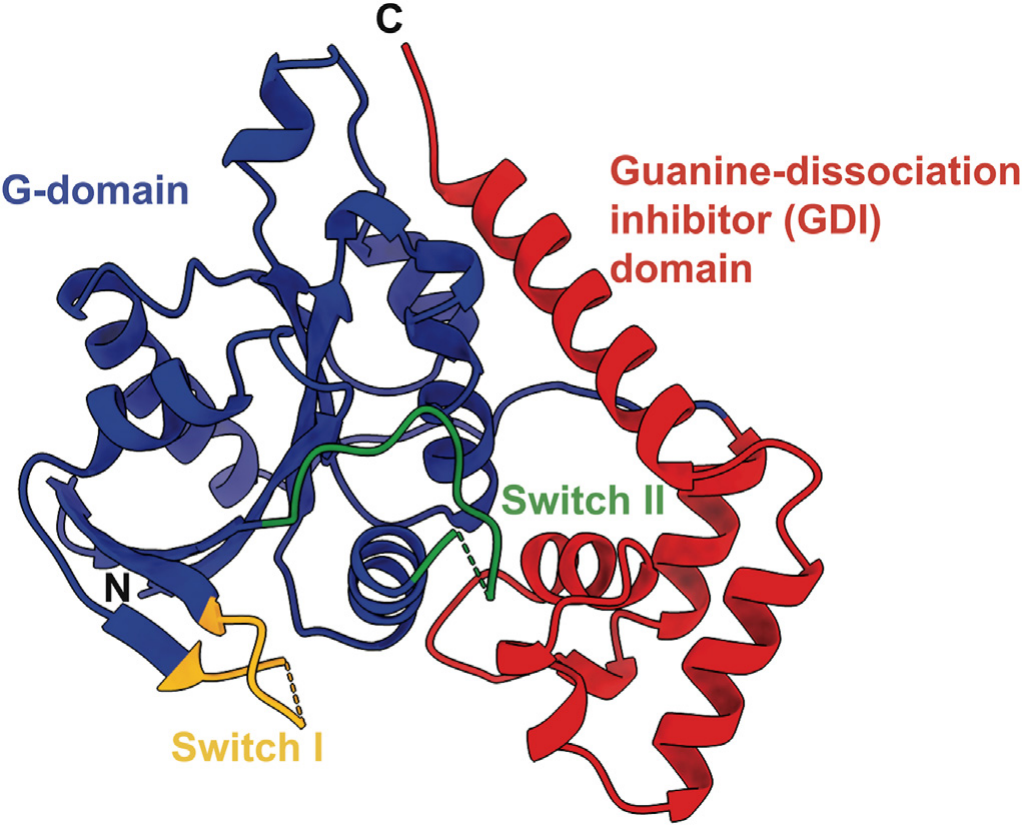
**X-ray crystal structure of the SUMO-cleaved *Vibrio cholerae* NFeoB NTPase domain in the *apo* state (PDB ID****9BA6****).** The overall structure of the *Vc*NFeoB has a typical NFeoB fold and comprises two major domains: the guanine-dissociation inhibitor (GDI) domain (labeled in *red*) that regulates GDP release and connects to the transmembrane region, and the G-protein domain (labeled in *blue*) that binds and catalyzes nucleotide hydrolysis. Within the G-protein domain are two key switch regions (Switch I and Switch II, labeled *yellow* and *green* respectively) that regulate nucleotide hydrolysis and transmit information to the GDI domain. In the absence of nucleotide, Switch I is mostly disordered, while Switch II is mostly ordered. ‘N’ and ‘C’ represent the N- and C-termini in the structure, respectively.

The X-ray crystal structure of the apo *Vc*NFeoB NTPase domain reveals the presence of a typical NFeoB fold (Fig. 2). Distinctly present are the two common NFeoB subdomains: the globular G-protein subdomain that is responsible for binding and hydrolyzing nucleotides (31) (Fig. 2, blue) and the hammer-shaped guanine-dissociation inhibitor (GDI) subdomain that regulates nucleotide dissociation (32) and connects directly to the FeoB transmembrane region (Fig. 2, red). Within the G-protein domain, two switch regions (known as Switch I and Switch II) that regulate nucleotide hydrolysis and communicate nucleotide status to the GDI domain (31, 32) are present, albeit Switch I is mostly disordered, while Switch II is mostly ordered in the apo form. Surprisingly, a comparison of the *Vc*NFeoB NTPase domain to other structurally characterized NFeoB domains displays strong structural conservation in both subdomains (C_α_ RMSD <1 Å on average) even though the NFeoB is not a strict GTPase (Fig. S2). These observations indicate that gross structural changes in the NFeoB region do not account for the observed nucleotide promiscuity of *V. cholerae* FeoB *per se*.

### The GDP-bound structure of the VcNFeoB NTPase domain reveals important nucleotide-binding residues

To determine whether structural properties within the nucleotide-binding pocket could contribute to nucleotide promiscuity, we sought to determine the structure of *Vc*NFeoB in the presence of various nucleotides. To do so, we co-crystallized apo *Vc*NFeoB (both SUMO-cleaved and (His)_6_ tagged) in the presence of hydrolyzed nucleotides (*e.g.,* ADP and GDP) and the presence of non- or slowly-hydrolyzable triphosphate mimics (*e.g.,* AMP-PNP, AMP-PCP, and GMP-PNP). Despite extensive fine screening and testing of multiple conditions, we were only able to generate datasets of GDP-bound *Vc*NFeoB(His)_6_ that diffracted modestly, but completely, to 4.2 Å resolution in the *C*_121_ space group consistent with 4 molecules in the asymmetric unit (ASU) (Table S1). This oligomerization is likely crystallization-induced based on our in-solution studies (Fig. S1). Using molecular replacement with our 2.3 Å apo *Vc*NFeoB structure coupled with strongly restrained refinement approaches, we built a model that clearly displayed GDP in the nucleotide-binding pocket of all molecules in the ASU based on omit maps (Fig. S3). Using the X-ray structure of GDP-bound *E. coli* NFeoB as a guide (PDB ID 3I8X; 2.3 Å resolution), we were able to place GDP in all *Vc*NFeoB molecules in the ASU and to solve this structure (*R*_w_/*R*_f_ = 0.223/0.273) (Table S1).

**Figure 3:**
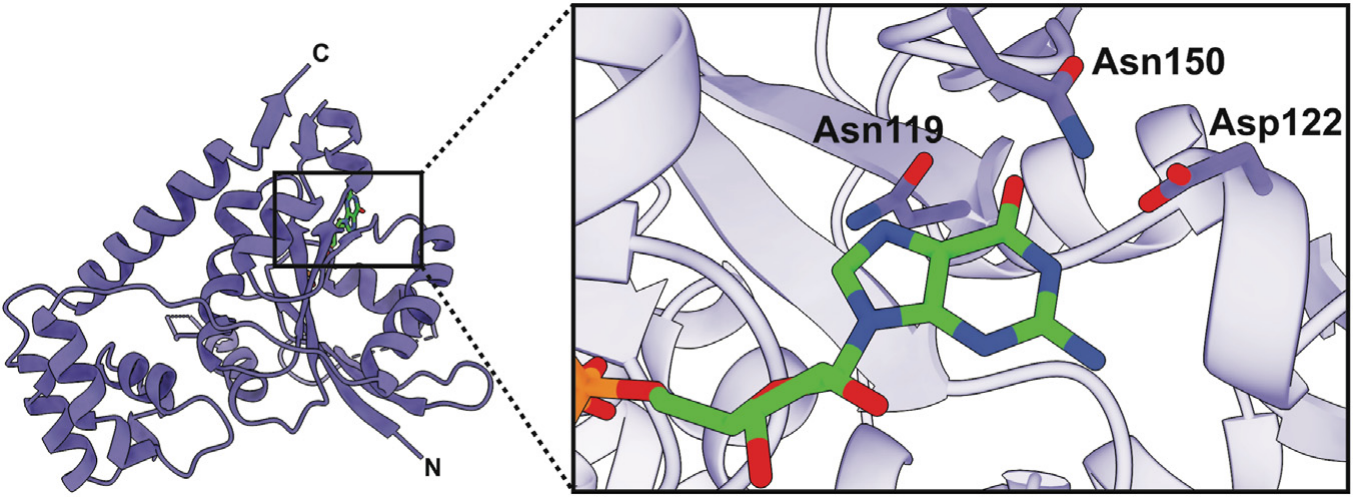
**X-ray crystal structure of the *Vibrio cholerae* NFeoB NTPase domain in the GDP-bound state.** The *right* panel represents a zoomed-in view of the nucleotide-binding pocket bound to GDP. Three residues make important contact with the purine ring: Asn119, Asp122, and Asn150. Of these residues, only Asn150 is located on a variable loop region (G5) and lacks conservation. In the presence of GDP, the Switch I region is nearly fully disordered, while the Switch II region is only partially disordered. ‘N’ and ‘C’ represent the N- and C-termini in the structure, respectively.

**Figure 4:**
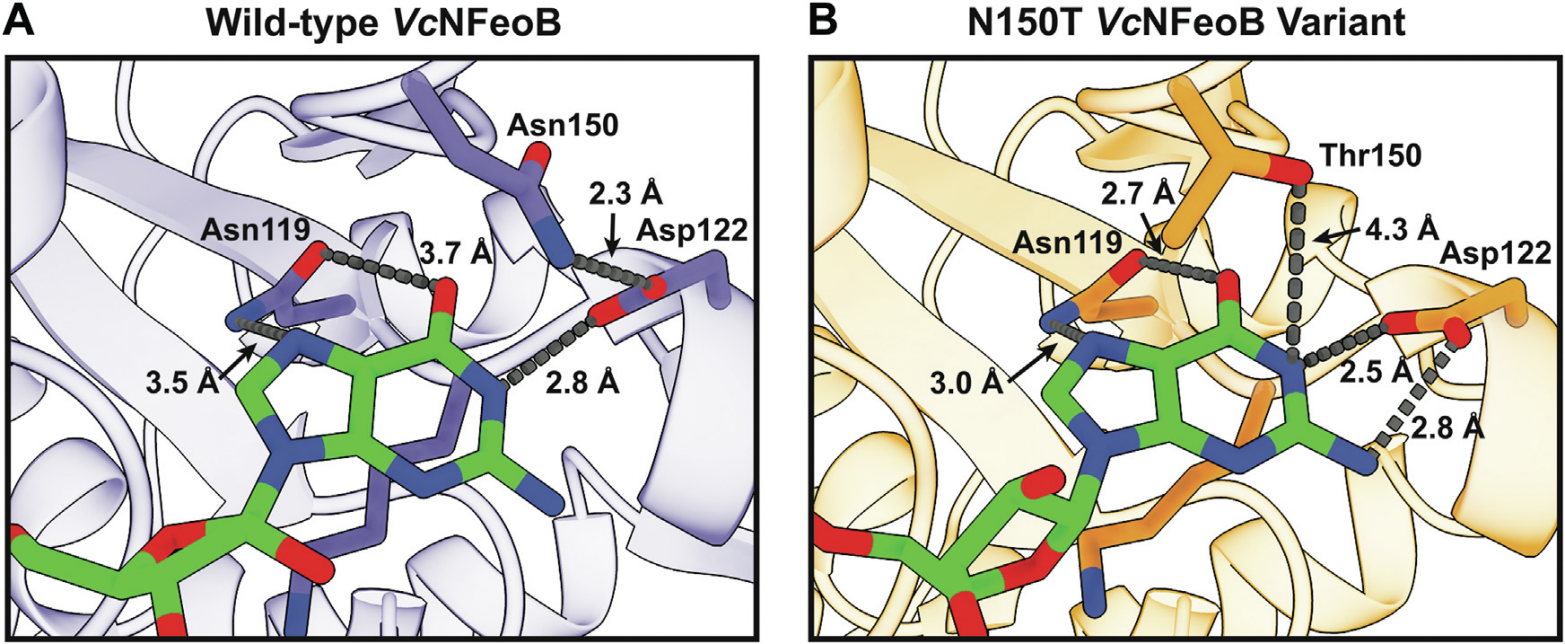
**Comparisons of thenucleotide-bindingpockets of the*****Vc*****NFeoB NTPase domain structures in theirGDP-boundforms.** Fewer hydrogen-bonding interactions are observed in the WT GDP-bound structure (*A*) compared to the N150T variant (*B*) of the NTPase domain. We hypothesize that the fewer hydrogen-bonding interactions in the WT structure allow for greater plasticity in NTP/NDP binding, unlike the strict GTPases that do not bind and hydrolyze other NTPs.

The crystal structure of *Vc*NFeoB(His)_6_ bound to GDP reveals multiple amino acids that contribute to nucleotide binding (Fig. 3). In the absence of nucleotide, the binding pocket is fairly open, while binding of GDP elicits a contraction surrounding the nucleobase with the associated amino acids responsible for nucleotide recognition coming together (Fig. S4). In this structure, the Switch I loop that is important for GTP hydrolysis (31) is mostly disordered, which may be attributed to the flexibility of this region when GDP is bound. As the nucleobase enters the binding pocket, a region of random coil from Asn119 to Asp122 tightens (Fig. S4), and both residues (conserved amongst NFeoBs) become within hydrogen-bonding distance (3.5 and 2.8 Å, respectively) of the guanine purine (Figs. 3 and 4*A*). Underneath the guanine purine is Lys120, also conserved in many NFeoBs, which appears to prop up the hydrolyzed nucleotide in the binding pocket; electrostatic contributions from this residue are not observed in our structure, and in some NFeoBs (like those in *S. thermophilus, K. pneumoniae, E. coli* and *Gallionella capsiferriformans,* and *Thermotoga maritima*) this residue corresponds to a non-polar amino acid, either a Met or an Ala (31, 33, 34, 35, 36). Interestingly, Asn150 is positioned along a region of random coil (known as the G5 motif) above the guanine purine but turned towards, and tightly hydrogen bonded with Asp122 on the G4 motif (2.3 Å distance) (Figs 3 and 4*A*). Because of the high variability within the G5 region, we sought to use bioinformatics to gain a better understanding of whether Asn150 might have an important role in nucleotide discrimination.

### Alterations in hydrogen bonding surrounding the nucleotide-binding pocket are likely linked to the nucleotide promiscuity of VcNFeoB

**Figure 5:**
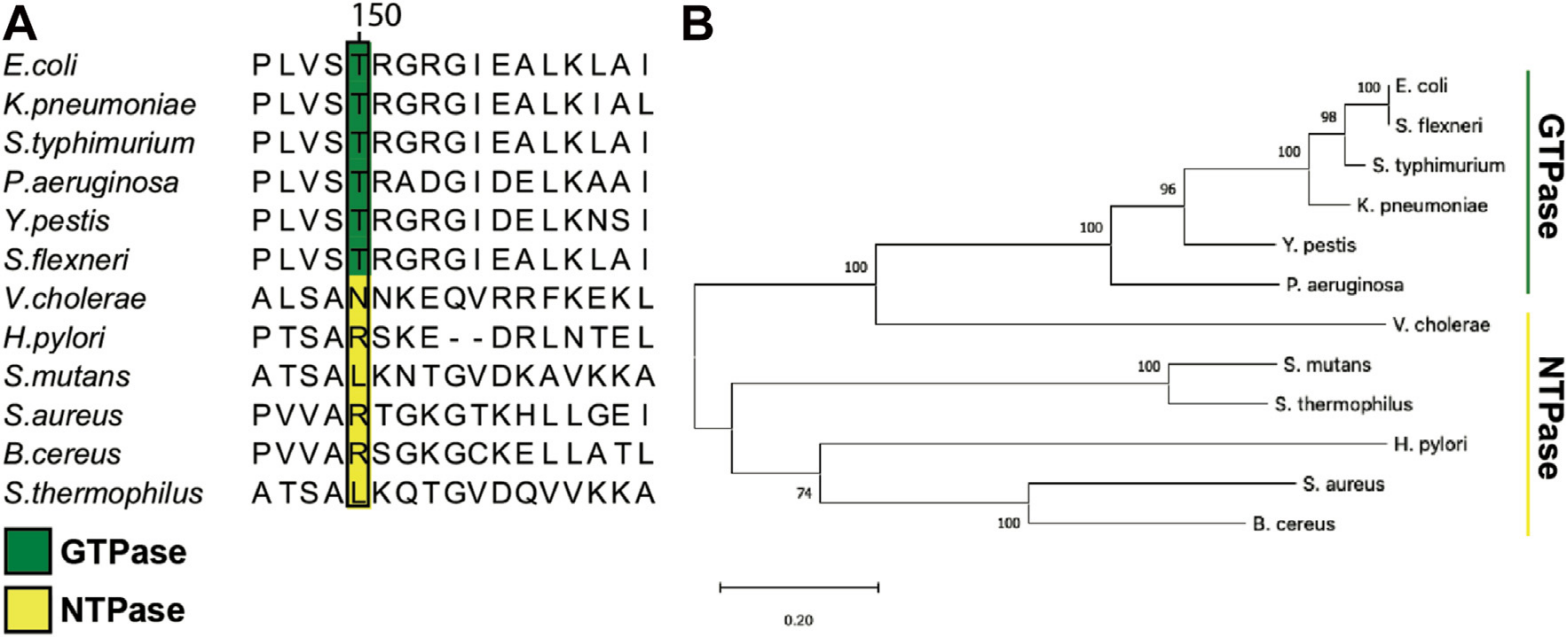
**Partial multiple sequence alignment (MSA) and phylogenetic analysis of nucleotide-specific and nucleotide-promiscuous FeoBs.***A*, the partial MSA compares residues in the variable G5 region of experimentally determined GTPases (*green*) and experimentally determined NTPases (*yellow*). At position 150 in *V. cholerae* FeoB (NTPase) is an Asn residue, while the analogous position 150 in the strict GTPase FeoBs is a Thr residue. The numbering above the MSA is based on the *V. cholerae* FeoB sequence. *B*, phylogenetic analysis of the FeoB sequences from the respective organisms in panel A shows distinct clustering of the NTPase FeoBs from those of the GTPase FeoBs. The 0.20 scalebar indicates the amount of genetic change at a certain length. The numbers above the nodes represent the bootstrap values above 50% from 500 iterations.

To gain a better understanding of residues that are either conserved or variable amongst NFeoB NTPases and GTPases, and to understand whether these sequence differences might contribute to functional differences, we used multiple sequence analysis (MSA) and phylogenetics. To do so, we limited our approach only to the sequences that have been previously tested *in vitro* to have either NTPase or strictly GTPase activities (28). Partial sequence alignments revealed that the position analogous to Asn150 within the G5 motif of the *Vc*NFeoB NTPase domain is highly variable for NFeoBs known to be NTPases, while this same position is an invariant Thr residue for NFeoBs known to be strict GTPases (Fig. 5*A*). The flanking regions of the G5 motif are also highly variable in NFeoB NTPases, while the same regions are highly conserved in NFeoB GTPases (Fig. 5*A*), suggesting that a degree of flexibility near the nucleobase may be important for nucleotide promiscuity. Intriguingly, phylogenetic analyses using the full-length sequences of *bona fide* FeoB NTPases and GTPases reveal a differential clustering among GTPase and NTPase proteins (Fig. 5*B*). While the number of FeoB proteins with verified nucleotide activity is low, this analysis is consistent with previous observations that suggest two different classes of NFeoBs may exist (28), which could be linked to the variability in the G5 motif and its flanking regions.

**Figure 6:**
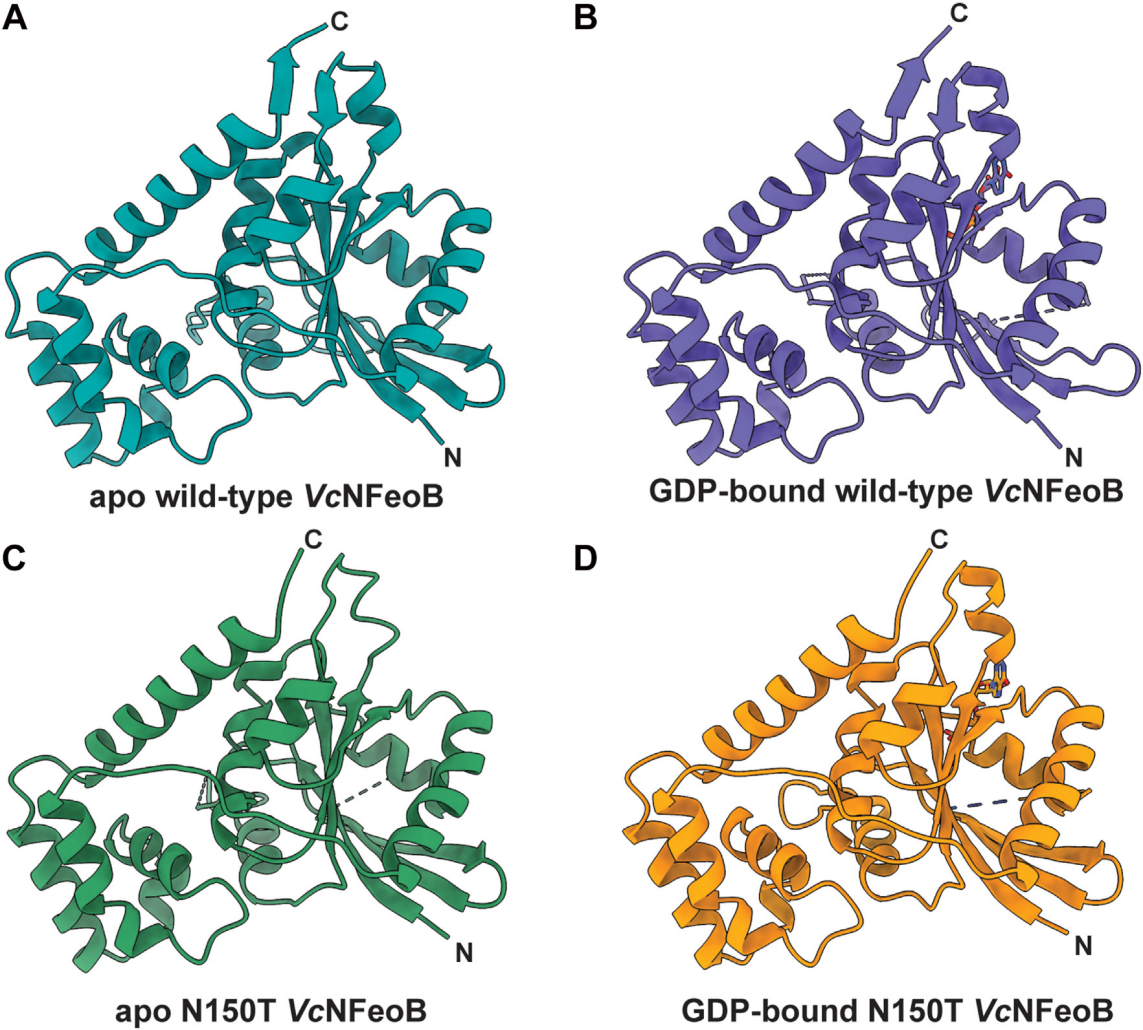
**Comparisons of the WT and N150T *Vc*NFeoB NTPase domain structures in their apo and GDP-bound forms.***A*, Apo WT *Vc*NFeoB(His)_6_ NTPase domain (PDB ID 8VWL). *B*, GDP-bound WT *Vc*NFeoB(His)_6_ NTPase domain (PDB ID 8VWN). *C*, Apo N150T *Vc*NFeoB(His)_6_ NTPase domain (PDB ID 9BA7). *D*, GDP-bound N150T *Vc*NFeoB(His)_6_ NTPase domain (PDB ID 9BA7). In general, the structural similarities among the WT and variant *Vc*NFeoB(His)_6_ NTPase domains are very high: 1.36 Å C_α_ RMSD (apo WT and apo N150T) and 0.88 Å C_α_ RMSD (GDP-bound WT and GDP-bound N150T). ‘N’ and ‘C’ represent the N- and C-termini in each structure, respectively.

To test the structural basis of this hypothesis, we expressed, purified, and crystallized the G5 motif N150T variant of *Vc*NFeoB(His)_6_ in the presence of GDP. The crystals diffracted to 2.9 Å resolution (Table S1), and initial analysis revealed one apo *Vc*NFeoB(His)_6_ NTPase domain and one GDP-bound *Vc*NFeoB(His)_6_ NTPase domain both within the asymmetric unit. This hetero-oligomerization is likely crystallization induced based on our in-solution studies (Fig. S1). Omit maps confirmed that substantial density was present and consistent with GDP in one, but not both, molecules within the asymmetric unit (Fig. S3), allowing us to visualize a direct comparison between the apo and the GDP-bound N150T variant at the same resolution (Fig. 6). Interestingly, the presence of Thr in position 150 affects the hydrogen bonding pattern surrounding the guanine nucleobase both directly and indirectly. First, the Thr hydroxyl moiety now makes a new hydrogen bond with position N1 along the purine ring (Fig. 4*B*). Second, the removal of Asn150 releases Asp122 to tighten and extend its hydrogen bonds with positions N1 and the H_2_N-C1 group (Fig. 4*B*). Third, Asn119 appears to pull the guanine further into the binding pocket, although uncertainty due to the modest resolution of the GDP-bound WT *Vc*NFeoB NTPase domain structure prevents a definitive statement regarding Asn119 hydrogen bonding strength. However, by comparison to the WT protein, the N150T variant appears to have two key additional hydrogen bonds surrounding the guanine purine that could affect nucleotide stability and may afford the discrimination of GTP relative to other NTPs. Finally, like the WT *Vc*NFeoB NTPase domain, the N150T variant displays a mostly disordered Switch I region and a partially disordered Switch II region in both the GDP-bound and apo forms.

### Isothermal titration calorimetry coupled with AlphaFold modeling reveals key differences in GTP/GDP and ATP/ADP binding

**Figure 7:**
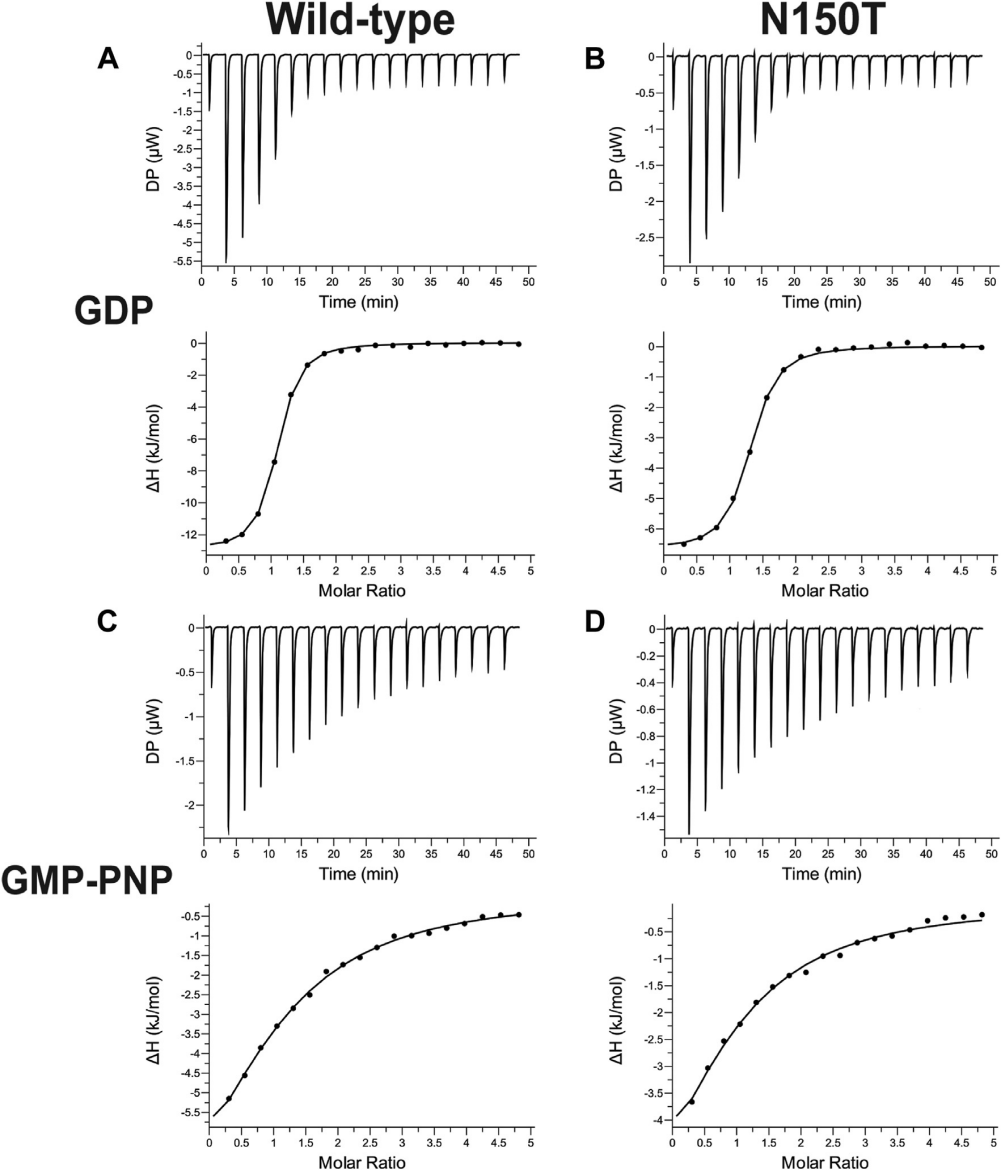
**The *Vc*NFeoB NTPase domain binds GDP and GMP-PNP similarly to GTP-specific NFeoB domains.** Representative ITC thermograms (*top*) and ΔH vs. molar ratio traces (*bottom*) of WT (*left*) and N150T (*right*) *Vc*NFeoB titrated with either GDP (*A*, *B*) or GMP-PNP (*C*, *D*). All datasets have been corrected for nucleotide dilution into buffer in the absence of protein. The GDP K_*d*_s for both WT (3.18 μM ± 0.23 μM) and N150T *Vc*NFeoB (3.60 μM ± 0.36 μM) are nearly identical, with both having a single binding site (N ≈ 1). The GMP-PNP K_*d*_ for WT *Vc*NFeoB (121.23 μM ± 34.13 μM) is slightly greater than the GMP-PNP K_*d*_ for N150T *Vc*NFeoB (93.95 μM ± 15.63 μM), but both reveal a single binding site (N ≈ 1). All values were determined in triplicate and represent the mean ± one standard deviation of the mean.

To test the binding strength and stoichiometry of various nucleotides to the WT *Vc*NFeoB NTPase domain and its N150T variant, we used isothermal titration calorimetry (ITC) (Fig. 7). Despite the hydrogen-bonding differences in our X-ray crystal structures, the WT and N150T *Vc*NFeoB proteins displayed nearly identical binding strengths to GDP: WT K_*d*_ of 3.18 μM ± 0.23 μM; N150T K_*d*_ of 3.60 μM ± 0.36 μM (Fig. 7, *A* and *B*) with a single binding site (N ≈ 1). By comparison to the binding of GMP-PNP (a non-hydrolyzable GTP analog), the WT and N150T *Vc*NFeoB constructs displayed slightly different binding strengths, consistent with our observed increase in hydrogen bonding in the N150T variant: WT K_*d*_ of 121.23 μM ± 34.13 μM; N150T K_*d*_ of 93.95 μM ± 15.63 μM (Fig. 7, *C* and *D*) with a single binding site (N ≈ 1). GDP is known to have a stronger affinity to NFeoBs than GTP/GMP-PNP due to the GDI domain (32, 37), and we observed a similar trend as our structural work also reveals the presence of a GDI domain in *Vc*NFeoB. Additionally, the GMP-PNP K_*d*_ values reported here are comparable to the enzymatic GTP-based K_M_s reported for WT- and N150T *Vc*NFeoB, suggesting a corollary between the K_*d*_ values determined in this work *via* ITC and those inferred based on activity assays (24). However, based on these data, the presence/absence of Asn150 alone does not dramatically change the binding strength of GTP/GDP to the *Vc*NFeoB NTPase domain. We then tested the ability of the *Vc*NFeoB NTPase domain to bind adenosine-containing nucleotides. While we attempted multiple different concentrations and stoichiometries using both hydrolyzed (ADP) and non-hydrolyzable analogs (AMP-PNP), we did not observe any appreciable saturation that could be fitted to any logical binding isotherm for both the WT and N150T variant proteins (Fig. S5). However, titrations of ADP/AMP-PNP showed strong amounts of heat evolution (up to 12 μW per injection) even when corrected for dilutions of nucleotide in the absence of protein (Fig. S5). These observations suggest either a rapid kinetic reversibility (*i.e.*, fast *k*_on_ and fast *k*_off_), and/or a potential conformational change that may be occurring as the domain samples ADP/AMP-PNP in the binding pocket.

Finally, as we were unable to crystallize *Vc*NFeoB bound to any adenosine nucleotides, we used the AlphaFold3 server to predict the structures of WT and N150T *Vc*NFeoB bound to ADP and ATP (Fig. S6). Interestingly, in the predicted WT structures in the presence of adenine-containing nucleotides, Asn150 makes a very weak interaction with the adenine purine while Asp122 and Ser148 (part of the G4 and G5 motifs, respectively) turn completely away from the binding pocket and make tight hydrogen-bonds (≈2.7 Å) with one another (Fig. S6*A*). A similar result was observed in the N150T variant with Thr150 making even weaker interactions with the adenine nucleotide (Fig. S6*B*), although it should be emphasized that these are both predicted models and not experimentally determined structures, and this minor hydrogen-bonding difference may not be meaningful. Notably, however, in all cases in which an adenine nucleotide is bound, strong hydrogen-bond interactions (≤2.8 Å) with Ser148 cause the NTPase domain to adopt an “Asp off” conformation in which Asp122 makes no interactions with the adenine nucleobase but instead turns away from the binding pocket. These results stand in contrast to the experimentally determined GDP-bound structures, as both adopt an “Asp on” conformation in which Asp122 makes strong interactions (≈2.4 Å–2.8 Å) with the guanine nucleobase in the binding pocket. When we compare the ADP/ATP-bound behavior to the apo structure of *Vc*NFeoB (Fig. S4), we note that the G4 and G5 regions would need to undergo structural rearrangements upon nucleotide binding to form the Asp “off” conformation, which could explain the strong heat evolution upon ADP and AMP-PNP titrations; however, fewer hydrogen bonding interactions likely preclude stable or prolonged binding within the pocket, explaining the weak binding observed in the ITC results. These data suggest that the nucleotide-promiscuous NFeoB domains likely use multiple residues to respond differently to the binding of guanine nucleotides compared to adenine nucleotides.

## Discussion

Whether FeoB, the primary prokaryotic ferrous iron transporter, is nucleotide promiscuous or nucleotide specific has vacillated for some time. Before the structure of NFeoB was known, early studies of FeoB predicted that the protein might hydrolyze ATP due to its sequence similarity to other ATPases, and decreased FeoB-dependent iron uptake in *H. pylori* was observed when ATP synthesis was disrupted by proton uncouplers (16, 24, 38, 39). However, subsequent studies of FeoB showed that the NFeoB domain from *E. coli* was GTP-specific (40), and structures of NFeoB from *Methanocaldococcus jannaschii* and *E. coli* revealed the presence of a G-protein like domain (34, 41), strongly implying that NFeoB bound and hydrolyzed only guanine nucleotides. This presumption continued for nearly two subsequent decades, as additional NFeoB structures were determined and FeoB was further explored in an almost GTP-exclusive manner (12, 14, 18). However, despite this assumption, Shin *et al.* reexamined NTPase activity in the context of *V. cholerae* FeoB and found this protein to be nucleotide promiscuous both *in vitro* and *in vivo* (24). In particular, two *Vc*NFeoB variants bearing altered residues along their G5 motifs, N150T and S148V, were notable in that they affected ATPase but not GTPase activity. These observations were further expanded to show that several bacterial FeoBs could be differentially classified as GTP-specific while others could be classified as nucleotide promiscuous (24, 25). However, the structural basis for this functional divergence of FeoB was not known.

In this work, we provided the first X-ray crystal structure of *V. cholerae* NFeoB, a notably promiscuous NTPase, in its WT and variant forms, both in the presence and absence of nucleotides. While the general NFeoB G-protein-like fold is conserved in our structures, the GDP-bound WT *Vc*NFeoB(His)_6_ structure revealed interactions between Asn150 (G5 motif) and Asp122 (G4 motif) that caused Asp122 to decrease the number of interactions it makes with the guanine nucleobase. As Asn150 is highly variable among NFeoB NTPases but conserved as a Thr among NFeoB GTPases, we wondered whether alteration of this residue alone could change nucleotide binding strength and/or specificity. Our structure of N150T *Vc*NFeoB(His)_6_ revealed increased H-bonding to the nucleobase due to the presence of Thr150 in the G5 motif, but this modification alone did not dramatically alter the binding affinity between NFeoB and GDP and only modestly increased the binding affinity between NFeoB and GMP-PNP. Instead, we hypothesized that the observed altered interactions might contribute more to nucleotide promiscuity as previously suggested based on activity analyses of *Vc*NFeoB variants (24).

While we were unable to determine experimental structures of adenine nucleotides bound to *Vc*NFeoB, ITC data couple with AlphaFold modeling provided insight into nucleotide promiscuity when taken in the context of our other experimentally-determined structures. For example, ITC analyses of ADP- or AMP-PNP titrated into *Vc*NFeoB revealed an isotherm that failed to saturate but displayed strong heat evolution even after correction for appropriate dilutions, distinct from the response of this domain in the presence of GDP and GMP-PNP. This unusual behavior could be interpreted to mean weak binding but also that conformational changes may accompany the interactions of ADP/AMP-PNP within the nucleotide-binding pocket, perhaps explaining why we failed to produce crystals of adenosine nucleotides bound to *Vc*NFeoB despite exhaustive trials. Structural modeling using AlphaFold supports this notion, as predicted structures of both ADP- and ATP-bound *Vc*NFeoB revealed movement of the G4 Asp122 and the G5 Ser148 residues, locking the two amino acids into a strong H-bond, and preventing Asp122 from interacting with the nucleobase (*i.e.*, “Asp off”). This conformational change opens up the binding pocket and results in only weak interactions as ADP/ATP enters, but then exits, the domain, likely rapidly. However, the rate of this conformational change must be slower than the rate of ATP hydrolysis, as we note that *Vc*NFeoB still hydrolyzes ATP under these conditions, albeit with a substantially attenuated rate based on *in vitro* activity assays of both N150T and S148V *Vc*NFeoB (24, 25). In fact, all three residues (Asp122, Ser148, and Asn150) combined displayed an important role for ATP hydrolysis (24), suggesting the G4 and G5 motifs are working in concert to contribute to nucleotide specificity, and prior activity data (24) support the conclusions from the structural and biophysical analyses presented in this work.

Interestingly, structural analyses of other bacterial NTPases reveal a similar amino acid pattern that may contribute to a more general nucleotide promiscuity (Fig. S7), which could be leveraged for a functional advantage by select organisms. For example, in the G5 motif position analogous to Asn150 in *Vc*NFeoB is a diverse set of amino acids that only interact with the nucleotide base weakly at best (24), but this weak interaction may be important for plasticity within the binding pocket. In contrast, the positions analogous to Asp122 and Ser148 in *Vc*NFeoB are conserved in these other bacterial NTPases. Conservation of Asp in this region is unsurprising, as the G4 NxxD motif is required for GTP hydrolysis (42), but the G5 Ser is not conserved; it may be possible that the Ser residue is needed to stabilize the “Asp off” conformation for NTPases to facilitate promiscuity in general, and observations that alterations of Ser148 decrease ATPase activity but not GTPase activity of *Vc*NFeoB support this hypothesis (24). Based on these observations, we propose that a combination of these three amino acids in G4 and G5 provide conformational flexibility to allow the utilization of both GTP and ATP for protein function.

The nucleotide promiscuity of FeoB likely has important mechanistic implications for the function of the transporter and may even provide an advantage to several bacterial pathogens that use this NTP-agnostic class of FeoBs. Regarding FeoB function, the ability of this conserved transporter to use GTP, ATP, and/or other NTPs for the function would ensure that Fe^2+^ acquisition remained functional even if the *in vivo* supplies of NTPs were disturbed during dyshomeostasis. As relatively few organisms maintain an alternative Fe^2+^ uptake system, the maintenance of Feo function is critical for bacterial ferrous iron acquisition. However, under homeostatic conditions, we propose that NTP promiscuous FeoBs are likely still GTP/GDP based on the stable and stronger interactions between FeoB and guanine-containing nucleotides observed in this study. Moreover, given the highly reactive nature of its translocated substrate, regulation of FeoB-mediated Fe^2+^ uptake by the GTP/GDP ratio could be an important protective mechanism to prevent iron overload within the cell. Nonetheless, given the critical nature of intracellular iron stores to metabolic function, the ability to acquire Fe^2+^ across a wide array of conditions could function as a virulence factor or another adaptive mechanism. Indeed, prior *in vivo* studies using a mutated strain of *V. cholerae* have demonstrated that ATPase-specific FeoB is sufficient to maintain a functional Feo iron transport system (24), suggesting that some organisms could leverage this function as an adaptive mechanism to ensure Fe^2+^ uptake regardless of the organism’s growth conditions. It is possible that this intriguing mechanism of FeoB function could be exploited to combat bacterial virulence in the future.

## Experimental procedures

### Cloning of NFeoB constructs

The *Vc*NFeoB(His)_6_ WT and N150T variant constructs were cloned into the pET-21a(+) plasmid as described previously (24) based on the sequence of WT *Vc*FeoB (Uniprot ID C3LP27). To create the N-terminal (His)_6_-SUMO-*Vc*NFeoB fusion, the gene encoding for *Vc*NFeoB was subcloned, and a synthetically added sequence for the Small Ubiquitin-like Modifier (SUMO) protein (Uniprot ID Q12306) was commercially appended (GenScript). The entire sequence was then subcloned into the pET-45b(+) plasmid between the *Pml*I and *Pac*I restriction sites, which allows for the translation of the N-terminal (His)_6_-SUMO-*Vc*NFeoB fusion when read in the frame.

### Expression of NFeoB constructs

The *Vc*NFeoB(His)_6_ WT and N150T variant plasmids were separately transformed into BL21(DE3) electrocompetent cells *via* electroporation, plated on Luria-Bertani (LB) plates supplemented with ampicillin (100 μg/ml), and incubated at 30 °C overnight. The next day, starter flasks containing 100 ml LB broth and ampicillin (100 μg/ml) were inoculated with a single colony each (WT and N150T) and allowed to grow overnight at 30 °C with 200 RPM shaking. The next day, 25 ml of the overnight cultures were inoculated into 1 L flasks charged with 1 L LB broth and ampicillin (100 μg/ml, final), and these cells were grown at 37 °C with shaking of 200 RPM. When OD_600_ reached 0.4 to 0.8, the cells in the flasks were then cold shocked at 4 °C for 2 h before induction with isopropyl β-D-1-thiogalactopyranoside (IPTG) to a final concentration of 1 mM. Cells were then grown for *ca.* 20 h overnight and harvested the next day by spinning at 5000*g*, resuspended in resuspension buffer (50 mM Tris pH 8.0, 300 mM NaCl, and 10% (v/v) glycerol) before being flash frozen on N_2*(l)*_ and stored at −80 °C.

All purification steps were conducted at 4 °C unless otherwise stated. Frozen cells were thawed and diluted to 100 ml with resuspension buffer, and 1 mM (final) phenylmethylsulfonyl fluoride (PMSF) was added prior to sonication at 80% amplitude, 30 s on pulse, 30 s rest pulse, 12 min total. Lysed cells were clarified by spinning at 163000*g* for 1 h. The supernatant was then applied to a 5 ml HisTrap HP column (Cytiva) that was pre-charged with Ni^2+^ and equilibrated with 5 column volumes (CVs) of wash buffer (50 mM Tris pH 8.0, 300 mM NaCl, 10% (v/v) glycerol and 1 mM TCEP). After the sample was applied, the column was washed with 10 CVs of wash buffer, then with wash buffer containing 50 mM imidazole, and eluted with wash buffer containing 150 mM imidazole. Eluted protein fractions were then pooled, and buffer was exchanged into ion exchange wash buffer (50 mM Tris pH 8, 10% (v/v) glycerol) using a 50 ml HiPrep 26/10 desalting column to remove all salt content before anion exchange chromatography. After desalting, the protein was then applied to a 5 ml HiTrap Q HP anion exchange column (Cytiva) that was then washed extensively with 10 CVs of the ion exchange buffer. The protein was purified *via* a linear elution gradient from 0 M to 1 M NaCl. Eluted protein fractions were pooled, concentrated *via* a 10 kDa molecular weight cutoff (MWCO) filter, and injected onto a 120 ml Superdex 75 preparative grade gel filtration column (Cytiva) after equilibration with 1.5 CVs of size exclusion buffer (25 mM Tris pH 8.0, 100 mM NaCl, 5% (v/v) glycerol and 1 mM TCEP). Fractions corresponding to pure, monomeric *Vc*NFeoB(His)_6_ were pooled and concentrated *via* a 10 kDa MWCO filter to *ca*. 12 mg/ml, aliquoted, flash frozen on N_2*(l)*_ and stored at −80 °C. An identical procedure was followed for the *Vc*NFeoB(His)_6_ N150T variant.

The cellular transformation, expression, cellular harvesting, cellular lysis, and initial Ni^2+^-based purification of the (His)_6_-SUMO-*Vc*NFeoB mirrored that of *Vc*NFeoB(His)_6_. After fractions were eluted from the 5 ml HisTrap HP column, the (His)_6_-SUMO-*Vc*NFeoB protein was buffer exchanged into SUMO cleavage buffer (50 mM Tris pH 8.0, 300 mM NaCl, 10% (v/v) glycerol, and 10 mM β-mercaptoethanol (BME)) and concentrated *via* a 10 kDa MWCO filter. House-made SUMO protease Ulp1 was then added at a 1:100 (mg/mg) ratio and allowed to cleave overnight with gentle rocking at 4 °C. The next day, the solution was applied again to a 5 ml HisTrap HP column to separate the now cleaved *Vc*NFeoB from any uncleaved protein. Eluted fractions containing cleaved *Vc*NFeoB were concentrated *via* a 10 kDa MWCO filter, injected onto a 120 ml preparative Superdex 75 column, eluted isocratically, pooled, and stored identically to *Vc*NFeoB(His)_6_ (*vide supra*).

### Crystallization of NFeoB constructs

Apo *Vc*NFeoB(His)_6_ was initially thawed and diluted to 10 mg/ml with size exclusion buffer prior to crystallization trials. Several commercial sparse-matrix screens were used to test for crystallization using vapor diffusion in sitting drop format. After incubation at 25 °C for *ca.* 4 months, crystals appeared in a condition containing 25% (w/v) PEG 3350, 0.1 M bis-Tris pH 5.5, and 0.2 M MgCl_2_. The crystals were then looped, cryo-protected, and frozen in N_2*(l)*_. Unfortunately, fine screens failed to replicate crystallization for further optimization.

To crystallize GDP-bound *Vc*NFeoB(His)_6_, protein at 10 mg/ml was incubated with 3 mM GDP for 2 h at room temperature before sparse-matrix screens were used to test for crystallization using vapor diffusion in sitting drop format at 25 °C. After 2 weeks, small, cubic-shaped crystals appeared in a condition containing 25% (w/v) PEG 3350, 0.1 M bis-Tris pH 5.5, 0.2 M MgCl_2_, and 0.1 M LiCl. Crystals were then looped, cryo-protected, and frozen in N_2*(l)*_.

The preparation of GDP-bound *Vc*NFeoB(His)_6_ N150T was identical to that of the WT protein. Sparse-matrix screens were used to test for crystallization using vapor diffusion in sitting drop format at 25ºC. Crystals initially appeared in conditions containing 24% (w/v) PEG 3350, 0.1 M bis-Tris pH 5.5, 0.03 M MgCl_2_, and 0.2 M (NH_4_)_2_SO_4_. Crystals matured after 2 weeks and were then looped, cryo-protected, and frozen in N_2*(l)*_.

To crystallize SUMO-cleaved *Vc*NFeoB, the protein was initially thawed and incubated with 3 mM ADP prior to dilution to 10 mg/ml. Sparse-matrix screens were used to test for crystallization using vapor diffusion in sitting drop format at 20 °C. After 2 weeks of incubation, clustered crystals appeared in a condition containing 30% (w/v) PEG 2000, 0.1 M Tris pH 8.0. Single crystals were separated manually using crystallization tools after the clusters were transferred to a drop containing cryo-protectant. Separated single crystals were then looped and frozen in N_2*(l)*_.

### X-ray diffraction, data reduction, and structural determination

Diffraction data were collected at the Advanced Photon Source (APS), Argonne National Laboratory on LS-CAT beamline 21-ID-D and at Brookhaven National Laboratory beamline 17-ID-2 (FMX). Data were automatically processed using Xia2 (43) and/or AutoProc (44). The initial phases of all datasets were determined by molecular replacement (MR) using Phenix Phaser (45) with an AlphaFold-generated model as an initial search input (46). After an initial MR solution was identified, further model building was accomplished using Phenix AutoBuild (45). The unambiguous presence of GDP in the nucleotide-binding site was confirmed by the generation of Polder maps (47) in Phenix for GDP-bound *Vc*NFeoB(His)_6_ WT and N150T datasets. The initial placement of GDP was determined based on the structure of *E. coli* NFeoB bound to GDP (PDB ID 3I8X) which was then further refined. Iterative rounds of manual model building and refinement were accomplished in Coot (48) and Phenix Refine (45), respectively, until model convergence and the final placement of any visible solvent molecules. Ramachandran statistics and clash values were determined from the MolProbity program (49) within the Phenix software suite. The following structures have been deposited in the Protein Data Bank: WT apo *Vc*NFeoB(His)_6_ (PDB ID 8VWL); WT GDP-bound *Vc*NFeoB(His)_6_ (PDB ID 8VWN); N150T GDP-bound *Vc*NFeoB(His)_6_ (PDB ID 9BA7); SUMO-cleaved apo *Vc*NFeoB (PDB ID 9BA6). Data collection and refinement statistics are provided for all structures in SI Table S1.

### Isothermal titration calorimetry

Purified WT or N150T *Vc*NFeoB(His)_6_ was diluted to 0.1 mM (3.2 mg/ml) in SEC buffer for all isothermal titration calorimetry (ITC) experiments. Experiments were conducted using the MicroCal PEAQ-ITC Automated instrument (Malvern Panalytical) to probe nucleotide binding. All titrations with nucleotide diphosphates (GDP and ADP) were performed in 25 mM Tris pH 8.0, 100 mM NaCl, 5% (v/v) glycerol, and 1 mM TCEP, while experiments involving the triphosphate mimics (GMP-PNP and AMP-PNP) were conducted using the same buffer conditions except with added MgCl_2_ to 10 mM final concentration. The calorimetry cell was loaded with 200 μl of WT or N150T *Vc*NFeoB(His)_6_, and 40 μl of nucleotide (GDP, GMP-PNP, ADP, or AMP-PNP) at 2.5 mM concentration (GDP and GMP-PNP) or 5.0 mM concentration (ADP and AMP-PNP) was loaded into the injection syringe. Thermal equilibrium was reached at 25 °C after an initial 60 s delay followed by 19 × 2 μl serial injections into the cell with 150 s interval delays between injection points with high spinning. Data were analyzed using the Malvern MicroCal PEAQ ITC analysis tool and fitted to a binding isotherm that has a single site using the following equations:

Equation 1:

$$\Delta Q\left(i\right)=Q\left(i\right)+\frac{dV_{i}}{V_{o}}\left[\frac{Q\left(i\right)+Q\left(i-1\right)}{2}\right]-Q\left(i-1\right)$$

Where the heat released, ΔQ(i), from the ith injection is represented by ΔQ(i).

Equation 2:

$$Q=n\Theta M_{t}\Delta HV_{0}$$

Where the total heat (Q) is related to the number of sites (n), the fractional occupation (Θ) the total free concentration of the macromolecule (*M*_t_), the molar heat of ligand binding (ΔH), and the volume determined relative to zero for the unbound species (V_0_).

### Bioinformatic analyses

Based on previous studies in which nucleotide promiscuity of NFeoB from multiple organisms was initially uncovered (28), sequences were obtained from the Uniprot database of intact FeoBs. A multiple sequence alignment was constructed through the EMBL MUSCLE program using default parameters (50). The resultant alignment was visualized *via* Jalview (51, 52, 53) and was then entered into the MEGAX software (54, 55, 56) for phylogenetic analysis using the maximum likelihood method and 500 bootstrap iterations with a minimum coverage of 95%. The final phylogenetic results were also visualized using MEGAX.

### Structural prediction using AlphaFold3

Predicted *Vc*NFeoB structures with adenosine nucleotides (ATP and ADP) were generated using the AlphaFold3 (57) server by submitting amino acids 1 to 261 from *Vc*FeoB (Uniprot ID C3LP27) with either ADP and Mg^2+^ or ATP and Mg^2+^ and utilizing the default parameters. In all cases, the lowest energy calculated structure is displayed as being representative, but the resulting five calculated structures for each prediction reveal very similar results. The calculated structures were both visualized and analyzed using ChimeraX (58).

## Data availability

All data are contained within the manuscript, either in the main body or in the Supplemental data submitted with the manuscript, and/or deposited in repositories such as the Protein Data Bank (PDB).

## Supporting information

This article contains supporting information.

## Conflict of interest

The authors declare that they have no conflicts of interest with the contents of this article.

## References

[1] Steimle S., van Eeuwen T., Ozturk Y., Kim H.J., Braitbard M., Selamoglu N. (2021). Cryo-EM structures of engineered active bc1-cbb3 type CIII2CIV super-complexes and electronic communication between the complexes. Nat. Commun..

[2] Booker S., Broderick J., Stubbe J. (1993). Ribonucleotide reductases: radical enzymes with suicidal tendencies. Biochem. Soc. Trans..

[3] Glorieux C., Calderon P.B. (2017). Catalase, a remarkable enzyme: targeting the oldest antioxidant enzyme to find a new cancer treatment approach. Biol. Chem..

[4] Einsle O., Rees D.C. (2020). Structural enzymology of nitrogenase enzymes. Chem. Rev..

[5] Hu Y., Ribbe M.W. (2015). Nitrogenase and homologs. J. Biol. Inorg. Chem..

[6] Schalk I.J., Mislin G.L.A. (2017). Bacterial iron uptake pathways: gates for the import of bactericide compounds. J. Med. Chem..

[7] Cain T.J., Smith A.T. (2021). Ferric iron reductases and their contribution to unicellular ferrous iron uptake. J. Inorg. Biochem..

[8] Choby J.E., Skaar E.P. (2016). Heme synthesis and acquisition in bacterial pathogens. J. Mol. Biol..

[9] Skaar E.P. (2010). The battle for iron between bacterial pathogens and their vertebrate hosts. PloS Pathog..

[10] Smith A.D., Wilks A. (2015). Differential contributions of the outer membrane receptors PhuR and HasR to heme acquisition in Pseudomonas aeruginosa. J. Biol. Chem..

[11] Cescau S., Cwerman H., Létoffé S., Delepelaire P., Wandersman C., Biville F. (2007). Heme acquisition by hemophores. Biometals.

[12] Brown J.B., Lee M.A., Smith A.T. (2021). Ins and outs: recent advancements in membrane protein-mediated prokaryotic ferrous iron transport. Biochemistry.

[13] Gómez-Garzón C., Barrick J.E., Payne S.M. (2022). Disentangling the evolutionary history of feo, the major ferrous iron transport system in bacteria. mBio.

[14] Sestok A.E., Linkous R.O., Smith A.T. (2018). Toward a mechanistic understanding of Feo-mediated ferrous iron uptake. Metallomics.

[15] Lau C.K., Krewulak K.D., Vogel H.J. (2016). Bacterial ferrous iron transport: the Feo system. FEMS Microbiol. Rev..

[16] Hantke K. (1987). Ferrous iron transport mutants in *Escherichia coli* K12. FEMS Microbiol. Lett..

[17] Sestok A.E., Brown J.B., Obi J.O., O'Sullivan S.M., Garcin E.D., Deredge D.J. (2022). A fusion of the Bacteroides fragilis ferrous iron import proteins reveals a role for FeoA in stabilizing GTP-bound FeoB. J. Biol. Chem..

[18] Sestok A.E., Lee M.A., Smith A.T., Hurst C.J. (2020). Advances in Environmental Microbiology: Microbial Metabolism of Metals and Metalloids.

[19] Brown J.B., Lee M.A., Smith A.T. (2022). The structure of Vibrio cholerae FeoC reveals conservation of the helix-turn-helix motif but not the cluster-binding domain. J. Biol. Inorg. Chem..

[20] Smith A.T., Linkous R.O., Max N.J., Sestok A.E., Szalai V.A., Chacón K.N. (2019). The FeoC [4Fe-4S] cluster is redox-active and rapidly oxygen-sensitive. Biochemistry.

[21] Weaver E.A., Wyckoff E.E., Mey A.R., Morrison R., Payne S.M. (2013). FeoA and FeoC are essential components of the Vibrio cholerae ferrous iron uptake system, and FeoC interacts with FeoB. J. Bacteriol..

[22] Faruque S.M., Albert M.J., Mekalanos J.J. (1998). Epidemiology, genetics, and ecology of toxigenic Vibrio cholerae. Microbiol. Mol. Biol. Rev..

[23] Stevenson B., Wyckoff E.E., Payne S.M. (2016). Vibrio cholerae FeoA, FeoB, and FeoC interact to form a Complex. J. Bacteriol..

[24] Shin M., Mey A.R., Payne S.M. (2019). FeoB contains a dual nucleotide-specific NTPase domain essential for ferrous iron uptake. Proc. Natl. Acad. Sci. U. S. A..

[25] Gómez-Garzón C., Payne S.M. (2020). FeoB hydrolyzes ATP and GTP. Metallomics.

[26] Luo M., Han Z., Huang G., Li R., Liu Y., Lu J. (2022). Structural comparison of unconventional G protein YchF with heterotrimeric G protein and small G protein. Plant Signal. Behav..

[27] Osaka N., Hirota Y., Ito D., Ikeda Y., Kamata R., Fujii Y. (2021). Divergent mechanisms activating RAS and small GTPases through post-translational modification. Front Mol. Biosci..

[28] Shin M., Park J., Jin Y., Kim I.J., Payne S.M., Kim K.H. (2020). Biochemical characterization of bacterial FeoBs: a perspective on nucleotide specificity. Arch. Biochem. Biophys..

[29] Shin M., Jin Y., Park J., Mun D., Kim S.R., Payne S.M. (2021). Characterization of an antibacterial agent targeting ferrous iron transport protein FeoB against *Staphylococcus aureus* and Gram-positive bacteria. ACS Chem. Biol..

[30] Hagelueken G., Hoffmann J., Schubert E., Duthie F.G., Florin N., Konrad L. (2016). Studies on the X-ray and solution structure of FeoB from Escherichia coli BL21. Biophys. J..

[31] Ash M.R., Maher M.J., Guss J.M., Jormakka M. (2011). A suite of Switch I and Switch II mutant structures from the G-protein domain of FeoB. Acta Crystallogr. D Biol. Crystallogr..

[32] Eng E.T., Jalilian A.R., Spasov K.A., Unger V.M. (2008). Characterization of a novel prokaryotic GDP dissociation inhibitor domain from the G protein coupled membrane protein FeoB. J. Mol. Biol..

[33] Hung K.W., Chang Y.W., Eng E.T., Chen J.H., Chen Y.C., Sun Y.J. (2010). Structural fold, conservation and Fe(II) binding of the intracellular domain of prokaryote FeoB. J. Struct. Biol..

[34] Guilfoyle A., Maher M.J., Rapp M., Clarke R., Harrop S., Jormakka M. (2009). Structural basis of GDP release and gating in G protein coupled Fe2+ transport. EMBO J..

[35] Deshpande C.N., McGrath A.P., Font J., Guilfoyle A.P., Maher M.J., Jormakka M. (2013). Structure of an atypical FeoB G-domain reveals a putative domain-swapped dimer. Acta Crystallogr. Sect F Struct. Biol. Cryst. Commun..

[36] Hattori M., Jin Y., Nishimasu H., Tanaka Y., Mochizuki M., Uchiumi T. (2009). Structural basis of novel interactions between the small-GTPase and GDI-like domains in prokaryotic FeoB iron transporter. Structure.

[37] Petermann N., Hansen G., Schmidt C.L., Hilgenfeld R. (2010). Structure of the GTPase and GDI domains of FeoB, the ferrous iron transporter of Legionella pneumophila. FEBS Lett..

[38] Kammler M., Schön C., Hantke K. (1993). Characterization of the ferrous iron uptake system of Escherichia coli. J. Bacteriol..

[39] Velayudhan J., Hughes N.J., McColm A.A., Bagshaw J., Clayton C.L., Andrews S.C. (2000). Iron acquisition and virulence in Helicobacter pylori: a major role for FeoB, a high-affinity ferrous iron transporter. Mol. Microbiol..

[40] Marlovits T.C., Haase W., Herrmann C., Aller S.G., Unger V.M. (2002). The membrane protein FeoB contains an intramolecular G protein essential for Fe(II) uptake in bacteria. Proc. Natl. Acad. Sci. U. S. A..

[41] Köster S., Wehner M., Herrmann C., Kühlbrandt W., Yildiz O. (2009). Structure and function of the FeoB G-domain from methanococcus jannaschii. J. Mol. Biol..

[42] Peterson Y.K., Hazard S., Graber S.G., Lanier S.M. (2002). Identification of structural features in the G-protein regulatory motif required for regulation of heterotrimeric G-proteins. J. Biol. Chem..

[43] Winter G. (2010). Xia2: an expert system for macromolecular crystallography data reduction. J. Appl. Crystallogr..

[44] Vonrhein C., Flensburg C., Keller P., Sharff A., Smart O., Paciorek W. (2011). Data processing and analysis with the autoPROC toolbox. Acta Crystallogr. D Biol. Crystallogr..

[45] Adams P.D., Afonine P.V., Bunkóczi G., Chen V.B., Davis I.W., Echols N. (2010). PHENIX: a comprehensive Python-based system for macromolecular structure solution. Acta Crystallogr. D Biol. Crystallogr..

[46] Jumper J., Evans R., Pritzel A., Green T., Figurnov M., Ronneberger O. (2021). Highly accurate protein structure prediction with AlphaFold. Nature.

[47] Liebschner D., Afonine P.V., Moriarty N.W., Poon B.K., Sobolev O.V., Terwilliger T.C. (2017). Polder maps: improving OMIT maps by excluding bulk solvent. Acta Crystallogr. D Struct. Biol..

[48] Emsley P., Cowtan K. (2004). Coot: model-building tools for molecular graphics Acta Crystallogr D. Biol. Crystallogr..

[49] Williams C.J., Headd J.J., Moriarty N.W., Prisant M.G., Videau L.L., Deis L.N. (2018). MolProbity: more and better reference data for improved all-atom structure validation. Protein Sci..

[50] Edgar R.C. (2004). MUSCLE: multiple sequence alignment with high accuracy and high throughput. Nucleic Acids Res..

[51] Troshin P.V., Procter J.B., Barton G.J. (2011). Java bioinformatics analysis web services for multiple sequence alignment--JABAWS:MSA. Bioinformatics.

[52] Troshin P.V., Procter J.B., Sherstnev A., Barton D.L., Madeira F., Barton G.J. (2018). JABAWS 2.2 distributed web services for Bioinformatics: protein disorder, conservation and RNA secondary Structure. Bioinformatics.

[53] Waterhouse A.M., Procter J.B., Martin D.M., Clamp M., Barton G.J. (2009). Jalview Version 2--a multiple sequence alignment editor and analysis workbench. Bioinformatics.

[54] Kumar S., Stecher G., Li M., Knyaz C., Tamura K. (2018). Mega X: molecular evolutionary genetics analysis across computing platforms. Mol. Biol. Evol..

[55] Stecher G., Tamura K., Kumar S. (2020). Molecular evolutionary genetics analysis (MEGA) for macOS. Mol. Biol. Evol..

[56] Tamura K., Stecher G., Kumar S. (2021). MEGA11: molecular evolutionary genetics analysis version 11. Mol. Biol. Evol..

[57] Abramson J., Adler J., Dunger J., Evans R., Green T., Pritzel A. (2024). Accurate structure prediction of biomolecular interactions with AlphaFold 3. Nature.

[58] Meng E.C., Goddard T.D., Pettersen E.F., Couch G.S., Pearson Z.J., Morris J.H. (2023). UCSF ChimeraX: tools for structure building and analysis. Protein Sci..

