# Supplementary material for: Structural determinants of Vibrio cholerae FeoB nucleotide promiscuity
Source: J Biol Chem. 2024 Aug 14;300(9):107663. doi: 10.1016/j.jbc.2024.107663 (PMC11406355; doi:10.1016/j.jbc.2024.107663)
Supplement: Supplementary Table S1 [file mmc1.docx]

|  | Apo WT*Vc*NFeoB(His)_6_ in *P_1_* space group (PDB ID 8VWL) | GDP-bound WT*Vc*NFeoB(His)_6_ (PDB ID 8VWN) | Apo WT*Vc*NFeoB in *P_121_* space group (PDB ID 9BA6) | Apo and GDP-bound N150T *Vc*NFeoB(His)_6_ (PDB ID 9BA7) |
| --- | --- | --- | --- | --- |
| **Data Collection** |  |  |  |  |
| Beamline | APS 21-ID-D | APS 21-ID-D | NSLS-II 17-ID-2 | NSLS-II 17-ID-2 |
| Wavelength (Å) | 0.97918 | 1.12706 | 0.97934 | 0.97934 |
| Space group | *P_1_* | *C_121_* | *P_121_* | *P_21212_* |
| Cell Dimensions |  |  |  |  |
| *a*, *b*, *c* (Å) | 48.35, 84.12, 158.03 | 194.27, 155.31, 50.92 | 53.96, 73.44, 63.03 | 202.46, 50.09, 62.25 |
| α, β, γ (˚) | 76.17, 83.91, 74.51 | 90.00, 95.88, 90.00 | 90.00, 94.25, 90.00 | 90.00, 90.00, 90.00 |
| Resolution (Å) | 77.59-3.67 | 60.53-4.25 | 43.41-2.38 | 33.94-2.88 |
| *R*_merge_ | 0.122 (1.185) | 0.184 (0.912) | 0.130 (0.951) | 0.155 (1.907) |
| *CC_1/2_* | 0.998 (0.792) | 0.983 (0.356) | 0.992 (0.521) | 0.997 (0.330) |
| *I/σ(I)* | 7.0 (0.8) | 6.9 (0.9) | 11.4 (1.6) | 8.8 (0.9) |
| Completeness (%) | 95.0 (88.9) | 98.8 (87.8) | 99.9 (99.0) | 99.4 (100) |
| **Refinement** |  |  |  |  |
| Resolution (Å) | 77.57-3.67 (3.80-3.67) | 50.65-4.25 (4.41-4.25) | 39.46-2.38 (2.47-2.38) | 33.94-2.88 (2.98-2.88) |
| No. reflections | 24,186 | 10,396 | 19,694 | 14,940 |
| *R*_work_ | 0.210 | 0.223 | 0.203 | 0.214 |
| *R*_free_ | 0.268 | 0.273 | 0.266 | 0.268 |
| No. atoms/molecules |  |  |  |  |
| Protein | 15,757 | 7,888 | 4,101 | 3,962 |
| Mg^2+^ | 6 | 3 | 0 | 0 |
| GDP | 0 | 4 | 0 | 1 |
| Waters | 0 | 1 | 92 | 14 |
| Glycerol | 0 | 0 | 7 | 0 |
| Cl^-^ | 0 | 0 | 2 | 5 |
| SO_4_^2-^ | 0 | 0 | 0 | 6 |
| Average B-factors (Å^2^) | 147.0 | 150.0 | 48.0 | 85.0 |
| R.m.s. deviations |  |  |  |  |
| Bond lengths (Å) | 0.003 | 0.003 | 0.009 | 0.009 |
| Bond angles (˚) | 0.62 | 0.76 | 1.70 | 1.16 |
| Ramachandran plot |  |  |  |  |
| Favored | 95.2 % | 95.7 % | 97.6 % | 96.7 % |
| Allowed | 4.6 % | 4.1 % | 2.5 % | 3.3 % |
| Outlier | 0.2 % | 0.2 % | 0 % | 0 % |

**Table S1.** Data collection and refinement statistics for apo WT*Vc*NFeoB(His)_6_ (*P*_1_ space group), GDP-bound WT *Vc*NFeoB(His)_6_, cleaved WT*Vc*NFeoB (*P*_121_ space group), and apo and GDP-bound N150T *Vc*NFeoB(His)_6_. Parentheses indicate the highest resolution shells.
